# Fungi from a Groundwater-Fed Drinking Water Supply System in Brazil

**DOI:** 10.3390/ijerph13030304

**Published:** 2016-03-09

**Authors:** Helena M.B. Oliveira, Cledir Santos, R. Russell M. Paterson, Norma B. Gusmão, Nelson Lima

**Affiliations:** 1 Department of Antibiotics, Federal University of Pernambuco, Av. Prof. Morais Rego, 1235, Recife, Pernambuco 50670-901, Brazil; helenambo@yahoo.com.br (H.M.B.O.); nbg@ufpe.br (N.B.G.); 2 Department of Chemical Sciences and Natural Resources, BIOREN-UFRO Scientific and Technological Bioresource Nucleus, Universidad de La Frontera, Temuco 4811-230, Chile; cledir.santos@ufrontera.cl; 3 Centre of Biological Engineering, University of Minho, Campus de Gualtar, Braga 4710-057, Portugal; russell.paterson@deb.uminho.pt

**Keywords:** filamentous fungi, yeasts, water distribution system, pathogens

## Abstract

Filamentous fungi in drinking water distribution systems are known to (a) block water pipes; (b) cause organoleptic biodeterioration; (c) act as pathogens or allergens and (d) cause mycotoxin contamination. Yeasts might also cause problems. This study describes the occurrence of several fungal species in a water distribution system supplied by groundwater in Recife—Pernambuco, Brazil. Water samples were collected from four sampling sites from which fungi were recovered by membrane filtration. The numbers in all sampling sites ranged from 5 to 207 colony forming units (CFU)/100 mL with a mean value of 53 CFU/100 mL. In total, 859 isolates were identified morphologically, with *Aspergillus* and *Penicillium* the most representative genera (37% and 25% respectively), followed by *Trichoderma* and *Fusarium* (9% each), *Curvularia* (5%) and finally the species *Pestalotiopsis karstenii* (2%). *Ramichloridium* and *Leptodontium* were isolated and are black yeasts, a group that include emergent pathogens. The drinking water system in Recife may play a role in fungal dissemination, including opportunistic pathogens.

## 1. Introduction

Fungi in water distribution systems are well known and have gained importance recently [1,2,3,4,5,6,7,8,9,10,11,12,13,14,15]. It is unsurprising that fungi are isolated from the surface or underground raw water in reservoirs and distribution systems [8,16,17,18] since they are found in almost every environmental niche. Fungi are also associated with the obstruction of water piping and the presence of odor and pigments in water [6,18]. Fungal contamination of water has major implications for hospitals and health institutions, with high concerns for immunocompromised individuals [10,15,19]. However, none of these authors has reported large water-borne mycotic outbreaks affecting consumers of water, although intense allergic responses from fungi in water have been recorded [15]. Various studies have demonstrated the relationship between water and microsporidiosis infection [20,21]. Apart from these, the lack of information on rapid poisonings or diseases of humans or other animals, associated with fungal occurrence in drinking water can be attributed to a lack of prolonged, systematic studies [6]. 

Water microbial standards in most countries are based on fecal bacteria as risk indicators for the presence of pathogenic bacteria and intestinal protozoa [22]. Thus only the oral-fecal route is taken in account which is not the main path for most fungal entry into water systems. Indeed, no correlation was found between (a) indicator bacteria such as *E. coli* and other coliforms and (b) filamentous fungi in a drinking water system [4].

Fungi from soil, air, crops, plant debris, organic matter, *etc.*, may enter the water systems in various ways, although water is regarded as an unnatural environment [10]. Fungi can survive and persist after treatment, or enter during installation, repairs, replacement of pipes and during depressurisation events, hence contaminating the water that reaches consumers [8,23,24,25]. The inhalation of spores after aerosolisation of water may occur, when water passes through taps and showers, which is a concern for health care institutions such as hospitals [10,26,27,28].

Fungi have been excluded from legislation on water quality except for a general all inclusive “mold count” in very few countries. World Health Organisation (WHO) does not recommend a limit for fungi in drinking water and lists bacteria, viruses, protozoa and helminths as the pathogens transmitted through drinking water [22,29]. In contrast, Sweden under the National Food Administration Regulation (guideline values for drinking water according to SLVFS 2001:30) established 100 colony forming units (CFU)/100 mL as limit to microfungi. A useful report written for the UK government on fungi in drinking water is available [30]. Also, several papers demonstrate pathogenic and opportunist fungi in drinking water [31]. Taylor *et al.* [32] identified 307 fungal species out of 1415 infectious organisms known to be pathogenic to humans. 

How best to treat fungi in drinking water has still to be established [30]. Before conclusions on health issues from fungi can be made, further studies are needed to assess the presence and consequences of the organisms in the drinking water environment [14]. This present report contributes to the knowledge of the diversity of fungi present in a water supply system in Brazil. 

## 2. Materials and Methods 

### 2.1. The Water Treatment System

The system studied has been in operation since 2006, and supplies the Federal University of Pernambuco—UFPE, University Campus of Recife, near to the NIATE—Exact Sciences and Nature Centre/Science Computing Centre. It consists of a raw water gathering box, tray aerators, decanter, three filters, pump house, chlorination system, the decanter sludge drying gantry and a laboratory for water quality monitoring. This system treats groundwater at 72.3 m^3^·water/h flow rate from 3 wells near to the Technological and Geosciences Centre (CTG): CTG 1 with 34.1 m^3^·water/h, CTG 2 with 23.9 m^3^·water/h and, CTG 3 with 19.8 m^3^·water/h low rates. Another independent well in the Campus is responsible to supply the university hospital (UH) with a flow rate of 12 m^3^·water/h.

In the UFPE system, water of the CTG wells is pumped to a raw water gathering box, and percolates through aerators to oxidize soluble metals such as iron and manganese. From the aerators, water moves by gravity to sedimentation tanks, where flocs formed by aeration can settle. Decanted water is collected in gutters and flows to upstream filters. After filtration, the water flows into the tank of the treatment plant. In the output of this, chlorination is applied, and the water is piped to an elevated reservoir from which it is distributed to other reservoirs at the university centers. When necessary, water receives pre-chlorination before it enters into the sedimentation tank. In the university hospital, before being stored in the reservoir, the well water passes through a treatment system, which is operated and managed separately by the hospital administration, and consists of a tray aerator and an activated carbon filter. 

### 2.2. Water Supplies

Because of high demand and the need for a continuous supply of water, the university hospital also uses water from the public water distribution system. In some extreme, scarce water situations at the university restaurant, water vender companies supply the system using a truck tanker.

### 2.3. Sampling Sites

Four reservoirs sites in the Federal University of Pernambuco Campus of Recife, were studied: a treatment plant (TP), a university restaurant (UR), the physical education center (PEC) and the university hospital (UH). 

### 2.4. Samples Collection

Water was collected in two different periods, from September 2013 to March 2014 and from January to July 2015, on a bimonthly basis, from four reservoir sites. Sterilized 1 L plastic bottles were used to collect the water according to the standard methods in [33]. Samples were transported and kept refrigerated until analysis in triplicate for physical, chemical and mycological characteristics.

### 2.5. Physical-Chemical Analysis of Water

The parameters residual free chlorine, temperature, pH, conductivity, turbidity, dissolved oxygen, and total organic carbon (TOC) were analyzed. The free residual chlorine was measured at the time of collection by visual comparison using a PD kit Hach model CN-70 (Hach Company Loveland, CO, USA), according to the manufacturer’s instruction. Temperature, pH, conductivity, turbidity, and dissolved oxygen, were determined at the time of collecting by a water quality multiparameter meter HORIBA U50 model (HORIBA Ltd, Miyanohigashi, Kisshoin Minami-ku, Kyoto, Japan). Aliquots from each sample were collected for TOC analysis at the Environmental Sanitation Laboratory of Technology and Geosciences Centre—UFPE. The 680 °C combustion catalytic oxidation method of the TOC-Vcsh analyzer (Shimadzu Corporation, Tokyo, Japan) was used.

### 2.6. Mycological Analysis

#### 2.6.1. Fungal Quantification

Fungi were quantified by the membrane filtration method [33] using cellulose ester membrane filters with 47 mm diameter and 0.45 μm pore size (code ME 25/21 ST; F. Maya Ind. Ltd., Cotia, SP, Brazil). From each sample, volumes of 30 and 100 mL were filtered in aliquots of 5 and 10 mL respectively, half of which was transferred to culture plates of R2A agar (Difco-Becton, Dickinson and Company, Sparks, MD, USA: 0.5 g yeast extract, 0.5 g protease peptone, 0.5 g casamino acids, 0.5 g dextrose, 0.5 g soluble starch, 0.3 g sodium pyruvate, 0.3 g dipotassium phosphate, 0.05 g magnesium sulphate, 15 g agar and 0.1 g chloramphenicol in 1 L of distilled water). The remainder was added to Sabouraud dextrose agar plates containing (10 g peptone, 40 g dextrose, 15 g agar and 0.1 g chloramphenicol in 1 L of distilled water, pH 5.4–5.8). The grown colonies were counted after 10 days of incubation and the results expressed as CFU/100 mL.

#### 2.6.2. Isolation

Fungi grown in plates were isolated initially onto potato dextrose agar (PDA, Merck Millipore Corporation., Darmstadt, Germany: 4 g potato infusion (infusion from 200 g potatoes), 20 g glucose, 15 g agar in 1 L of distilled water) followed by cultivation in specific media to defined taxonomic groups at 25 °C after 7 days growth.

#### 2.6.3. Identification

Identification to species level was done phenotypically based on macroscopic and microscopic morphological features of cultivation in malt extract agar (MEA, Merck Millipore Corporation: 20 g malt extract, 1 g peptone, 20 g glucose, 15 g agar in 1 L of distilled water), Czapek yeast extract agar (CYA, 30 g sucrose, 3 g sodium nitrate, 1 g dipotassium phosphate, 0.5 g magnesium sulphate, 0.5 g potassium chloride, 0.01 g ferrous sulphate, 0.5 g yeast extract and 15 g agar in 1 L of distilled water), Czapek Dox solution agar (CZ, 30 g sucrose, 3 g sodium nitrate, 1 g dipotassium phosphate, 0.5 g magnesium sulphate, 0.5 g potassium chloride, 0.01 g ferrous sulphate and 15 g agar in 1 L of distilled water).

Incubation was at 25 °C and the characteristics, such as colony features in specific media, presence of dimorphism, size of conidia, *etc.*, were observed in a light microscope Leica DMR, and compared with those described in the literature [34,35,36]. The identifications were undertaken by trained fungal taxonomists at the institutes listed in the addresses.

## 3. Results and Discussion

### 3.1. Physical-Chemical Analysis

The water samples from TP were obtained before chlorination because the final chlorination is added to the reservoir outlet at the concentration of 2 mg/L. The PEC reservoir presented no residual chlorine (Table 1). Various factors could have contributed to this result: ineffective disinfection including the effect of particulate matter on the residual disinfectant. In fact, this sampling site presented the highest values of turbidity, ranging from 7.9 to 16.5 TU (turbidity units), contributing to disinfectant depletion. The UR and UH use a supplementary manual addition of chlorine in the reservoirs, however this is discontinuous and could not maintain the constant level of residual disinfectant. For this, when the pumps are not working properly or some failure occurs in the system, the procedure is to pour directly into the water chlorine solution. For UR, sodium dichloro isocynurate tablets are also used to keep the residual chlorine concentration between 0.2–2.0 mg/L. Values of residual chlorine ranged from 0.2 to 0.5 mg/L and are, in general, considered satisfactory for water disinfection of free-living microorganisms [29], however, in the present study, no chlorine was obtained in 22 out 24 samples for UR, PEC and UH.

The water temperatures ranged from 26.4 to 28.0 °C, with higher values in January 2014. The average value over the sampling period was 26.9 °C. 

The pH varied between 4.1 and 7.3 with a mean of 6.0. The highest occurred in the PEC reservoir in September 2013. These pHs were within the range favorable to the growth of fungi.

The water turbidity in all reservoirs varied between 5.2 and 16.5 TU. In the TP reservoir, values ranged from 5.7 to 13 TU and are much higher than the water potability standard for post filtration groundwater or pre-disinfection of 1.0 UT [37]. In the other sample sites, turbidity levels exceeded the maximum value of 5.0 UT at all points of the distribution network established by the water potability Brazilian standard [37]. The presence of suspended material and chlorine-demanding solutes in water reduce the ability of disinfection to inactivate microorganisms [29]. 

The conductivity of water values varies between 0.26 and 0.42 mS/cm (Table 1). The highest value occurred in the ETA tank in September 2013.

The dissolved oxygen content in samples ranged between 5.38 and 18.23 mg/L with a mean of 11.7 mg/L. These results show that the dissolved oxygen availability is not a limiting factor to the presence of aerobic microorganism, such as fungi, in the system and its concentration could be related to the water aeration performance on the tray aerators.

The TOC concentrations varied from 2.8 to 4.2 mgC/L, with an average of 3.37 mgC/L. The contents were slightly higher in March 2013. TOC is used to characterize the dissolved organic matter suspended in natural water, and the normal value of groundwater range 0.1–4 mgC/L [38]. The results show that the system provides water with normal organic carbon concentrations and imply that these are sufficient for fungal growth, especially for the oligotrophic species.

Overall, the range of the water physical-chemical parameters found can support a wide fungal diversity. 

### 3.2. Quantification of Fungi

**Figure 1:**
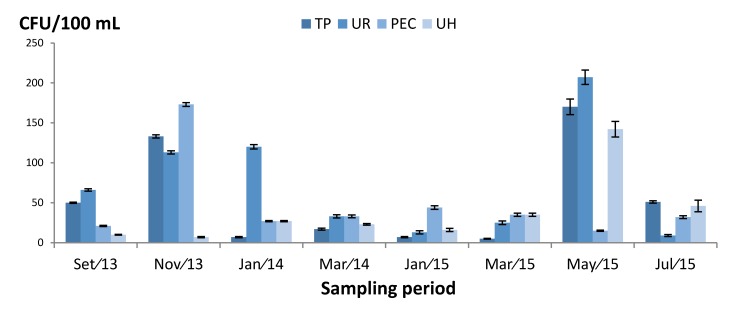
Quantification of water fungi express in colony forming units (CFU)/100 mL in different sampling sites and period. TP: Water treatment plant; UR: Univ. restaurant; PEC: Physical education center; UH: Univ. hospital.

All sampling sites were positive for filamentous fungi. In total, 1712 CFU were counted. Fungal counts ranged from 5 to 207 CFU/100 mL, with an average of 53 CFU/100 mL per sampling site (Figure 1). The highest counts occurred in the UR reservoir (15 May/2015) of 207 CFU/100 mL, followed by PEC (Nov/2013) with 173 CFU/100 mL. The lowest values occurred in the TP reservoir (Mar/2015) of 5 CFU/100 mL. 

Taking the Swedish legislation of 100 CFU/100mL for fungi in water as reference, 22% of the samples exceeded this limit. The UR sampling site had 207, 120 and 113 CFU/100 mL. The TP site exceeded twice with 170 and 133 CFU/100 mL. The PEC and UH exceeded once with each presenting 173 and 142 UFC/100 mL, respectively.

Filamentous fungi in drinking water are common in water distribution systems and can occasionally be isolated in high concentrations [10]. In Poland, the samples count ranged from 20 to 500 CFU/100 mL [17] and in Australia counts were 33 and 97 CFU/100 mL for the water from mains and reservoirs, respectively [22]. In Egypt, Samah *et al.* [39] analyzed ground water for filamentous fungi and found 4 to 119 CFU/100 mL, while in untreated ground water, an average of 66 CFU/100 mL were found [11]. Hence, many of the present values for Brazil are very high and may represent a health risk to consumers. 

Evidence suggests that fungi survive and multiply in distribution systems in biofilms and sediments, particularly at warmer temperatures, or where the flow is restricted [16]. An important aspect in the current work is that the water is stored. Reservoirs have features such as darkness, long retention times and stagnation zones that favor the proliferation of microorganisms and biofilm formation. Biofilms protect microorganisms from disinfection and can be responsible for transferring fungi (and other microorganisms) to the bulk of the water [13,40]. The physical-chemical water quality such as temperatures, turbidity and availability of TOC may encourage fungal growth. In addition, disinfection was demonstrated as deficient or missing in the present system.

The UH site, except samples taken on May 2015, presented the lowest numbers with a maximum of 46 and minimum of 7 CFU/100 mL. These results could be attributed to a more systematic application of chlorine, in addition to more intense surveillance and preventative actions contributing to fewer fungi.

### 3.3. Fungal Identification

Among 859 strains identified, the most abundant genera were *Aspergillus* (37%), *Penicillium* (25%), *Trichoderma* and *Fusarium* (9% each), and *Curvularia* (5%). *Acremonium*, *Cladosporium*, *Cunninghamella*, *Humicola* and *Leptodontium* were 1% while the remainder was less than 1%.

The fungi that were identified to species are showed in Table 2. These included *Aspergillus alliaceus*, *A. chevalieri*, *Aspergillus flavus*, *A. parasiticus*, *A. fumigatus*, *A. neoniveus*, *A. niger* complex (which can also include *A. amawori* and *A. tubingensis*), *A. terreus* and *A. versicolor*; *Penicillium citrinum*, *P. janczewskii*, *P. oxalicum* and *P. waksmanii*; *Fusarium solani*; *Trichoderma harzianum* and *T. viride*; *Curvularia pallescen*; and *Pestalotiopsis karstenii* which had a high frequency of 2%. Detected in small numbers were: *Cladosporium cladosporioides*, *Lichtheimia hyalospora*, *Paecilomyces variotii*, *Ramichloridium matsushimae*, *Scolecobasidium humicola*, and *Talaromyces purpurogenus*.

*Aspergillus*, *Penicillium*, *Fusarium*, *Trichoderma*, *Curvularia* and *Pestalotiopsis* were detected in all sampling sites. Among the *Aspergillus* species, *A. flavus* had the highest frequency, followed by *A. niger* complex, *A. parasiticus* and *A. versicolor*. *Penicillium citrinum* was the most abundant species within the penicillia. The species *A. flavus*, *P. citrinum* followed by *A. niger* complex had the highest frequency (respectively, 7.3%, 6.8% and 5.5%) in the sampling site TP, which can be attributed to the lack of chlorination. For PEC, where the chlorination was poor, *A. flavus* remained abundant (3.4%). In addition, *P. citrinum* shows resilience to the chlorination effect in the sampling sites UR (5.7%) and UH (5.2%) when compared with TP (6.8%). In contrast, the PEC sampling site shows no residual chlorine and the highest turbidity which is favourable to the fungi, but only 2.7% *P. citrinum* was obtained which means other external factors may be detrimental for this species.

The incidence of nosocomial fungal infections has dramatically increased in recent decades. *Aspergillus* is the second most frequent cause of nosocomial fungal infections, and aspergillosis tends to occur in immunocompromised patients [28,41]. Anaissie *et al.* [42] recovered *Aspergillus* species from a hospital water system and demonstrated the highest airborne *Aspergillus* propagule density (2.95 CFU/m^3^) in water used in bathrooms. In addition, they reported that water from tanks yielded higher colony-forming units than municipal water. Furthermore, many areas of the world demonstrate a wide distribution of fungi in water supplies [28]. About 4.8 million adult people worldwide who have asthma also have allergic bronchopulmonary aspergillosis. Of these 400,000 are estimated also have chronic pulmonary aspergillosis [43,44].

The present results show *A. fumigatus* appeared in three important sampling sites: TP, UR and UH. This fungus is a highly significant pathogen causing fungal infection in immunocompromised patients [28,45]. However, *A. fumigatus* was recovered from 49% of taps at Rikshospitalet University Hospital, Oslo [27] and a high occurrence of this species was found in domestic wells in Brazil [28]. Among invasive fungal infections, *A. fumigatus* accounts for 90% of cases and, *Fusarium* and zygomycetes are common problems amongst the remainder [6,31].

In this current work, all sampling sites presented *A. flavus* and *A. parasiticus* and these species are recognized producers of aflatoxins. Paterson *et al.* [46] demonstrated production of aflatoxins in a cold water storage tank. Water subjected to storage (e.g., reservoirs, cisterns and bottles) for long periods, may contain increased mycotoxin concentrations. The long term, daily consumption of large amounts of water containing low levels of mycotoxin, requires further consideration as a health hazard [10,15,30].

Interestingly, *P. citrinum* also occurred in all samples sites. This species produces the mycotoxin citrinin and is also implicated as a cause of mycoses [47]. The other penicillia in Table 2 have not yet been implicated in human mycoses. Ma *et al.* [48] analyzed a hospital hot water system for potentially pathogenic fungi using ITS sequencing and penicillia were the most abundant. In general, these fungi are minor contributors to human diseases, apart from *Talaromyces marneffei* which causes a lethal systemic infection (penicilliosis) [47]. Accordingly, it is very important to identify penicillia to the species level.

*Fusarium* and *Trichoderma* shared the same relative frequency in all samples. *Fusarium* can cause several opportunistic mycoses, such as subcutaneous infections and invasive mycoses in immunocompromised [49]. *F. solani* was isolated frequently from the sampling sites (Table 2) and is associated with the production of the water soluble T-2 toxin [50]. The *F. oxysporum* species and *F. solani* species complexes are responsible for 80% of human *Fusarium* infection [51]. They are able to form biofilms on contact lens and polyvinyl chloride pipes [52]. Related keratitis outbreaks associated with contact lens use were caused by *Fusarium* spp. in Southeast Asia and North America [53]. Water in plumbing systems was suggested as the main environmental reservoir for fusaria eye infections [54]. 

*Trichoderma* have been mentioned as emergent pathogens in association to risk factors such as peritoneal dialysis, organ transplantation, and hematologic disorder. Some species of this genera, such as *Trichoderma longibrachiatum*, which is mentioned as the main human pathogen of the genus, and *T. atroviride*, *T. citrinoviride*, *T. harzianum*, *T. koningii*, *T. orientale*, *T. pseudokoningii*, *T. reesei*, and *T. viride* are associated with infections, allergic sinusitis, keratitis, otitis, superficial and subcutaneous infection, peritonitis, endocarditis, brain abscess and deep pulmonary infection [55,56].

*Ramichloridium matsushimae* was found at a low percentage. This is a black yeast, a term used to describe melanised fungi that display a yeast state especially in culture. This fungus is an opportunistic human pathogen [57].

Finally, the R2A and Sabouraud media were not selective in separating clearly fungal taxa. This could be explained by the fungi not being under stress conditions due to the low or zero residual chlorine and the presence of considerable amounts of dissolved oxygen and organic matter in water samples. Furthermore, Sabouraud, as a richer medium, gave better conditions for the water fungi (conidia and propagules) to grow, which is reflected by the higher CFU obtained, of 15%–30% more when compared to R2A medium.

## 4. Conclusions 

Fungi occurred in all the reservoirs examined and often at high concentrations. Some of them are considered to produce mycotoxins and/or are opportunistic human pathogens. Water storage generates stagnation, stratification, particle accumulation, dead zones, depletion of residual disinfectant, and biofilm formation. These parameters combined with chemical-physical characteristics of the system water (high turbidity and temperature, pH, TOC and dissolved oxygen), are favorable to microbial growth making reservoirs a potential high risk of water quality degradation by fungi. 

Special attention should be given to reservoirs where local water is kept and where there is a risk of degradation of its quality. Frequent surveillance for fungi and the setting of limits are required to improve the mycological quality of drinking water.

## Figures and Tables

**Table 1:** Water physical-chemical data from the sampling sites with the minimum and maximum values obtained from the collection period.

Parameters	TP		UR		PEC		UH	
	Min	Max	Min	Max	Min	Max	Min	Max
Residual chlorine (mg/L)	-	-	0.0 *	1.5 *	0.0	0.0	0.0 *	1.0 *
Temperature (°C)	26.4	28.0	26.5	27.6	26.6	27.2	26.3	27.5
pH	5.0	6.6	4.1	6.4	5.1	7.3	5.6	6.7
Turbidity (UT)	5.7	13.0	7.5	13.6	7.9	16.5	5.2	8.3
Conductivity (mS/cm)	0.3	0.4	0.3	0.3	0.3	0.3	0.3	0.4
Dissolved oxygen (mg/L)	5.4	12.3	9.2	13.2	8.1	12.6	8.2	18.2
Total organic carbon (mgC/L)	2.8	3.6	2.8	4.2	2.9	4.2	3.7	4.0

TP: Water treatment plant; UR: Univ. restaurant; PEC: Physical education center; UH: Univ. hospital. -: chlorination is made after this point; *: supplementary chlorination is made manually.

**Table 2:** Relative frequency distribution of fungal isolates from water in the sampling sites.

Fungi	TP	UR	PEC	UH
	Relative frequency (%)			
*Aspergillus alliaceus*	0.2	0.1	0.6	
*Aspergillus chevalieri*		0.1		
*Aspergillus flavus*	7.3	0.1	3.4	2.7
*Aspergillus fumigatus*	0.2	0.2		0.2
*Aspergillus neoniveus*			0.2	
*Aspergillus niger* complex	5.5	1.4	1.3	0.8
*Aspergillus violaceofuscus*			0.2	
*Aspergillus parasiticus*	1.7	1.6	1.9	0.7
*Aspergillus terreus*	0.6	0.4		1.4
*Aspergillus versicolor*	0.2	3.3	0.6	0.7
*Penicillium citrinum*	6.8	5.7	2.7	5.2
*Penicillium corylophilum*				0.2
*Penicillium janczewskii*		0.9		0.5
*Penicillium janthinellum*				0.1
*Penicillium oxalicum*	0.1	1.0	0.8	
*Penicillium waksmanii*		0.1	1.0	
*Acremonium* sp.			0.4	1.0
*Chaetomium* sp.	0.2			
*Cladosporium cladosporioides*		0.9		0.1
*Cladosporium macrocarpum*		0.4		
*Colletotrichum* sp.		0.1		
*Cunninghamella* sp.		0.9	0.2	
*Curvularia pallescens*	1.3	2.0	0.2	1.8
*Fusarium solani*	0.7	2.2	1.4	4.5
*Humicola grisea*			0.6	
*Humicola fuscoatra*		0.1		
*Leptodontium* sp.	0.4	0.6		
*Lichtheimia hyalospora*		0.2		
*Myrothecium* sp.				0.1
*Paecilomyces aerugineus*		0.1		
*Paecilomyces variotii*		0.4		
*Pestalotiopsis karstenii*	1.0	0.1	0.7	0.5
*Phaeoacremonium* sp.	0.2	0.1		
*Phialophora richardsiae*	0.1			
*Phoma leveillei*				0.1
*Ramichloridium matsushimae*		1.0	0.1	
*Scolecobasidium humicola*			0.1	
*Talaromyces purpurogenus*			0.1	
*Trichoderma aureoviride*			0.7	
*Trichoderma harzianum*	0.6	2.6	1.0	3.1
*Trichoderma viride*			1.4	
*Verticillium* sp.	0.4			
Unidentified arthrosporic fungi	0.7	0.7	0.2	0.5

TP: Water treatment plant; UR: University restaurant; PEC: Physical education center and UH: University hospital.
